# Sleeping but not defenceless: seed dormancy and protection

**DOI:** 10.1093/jxb/erae213

**Published:** 2024-05-17

**Authors:** Benjamin Hubert, Olivier Leprince, Julia Buitink

**Affiliations:** INRAE, Institut Agro, Université d'Angers, IRHS, SFR QUASAV, F‐49000 Angers, France; INRAE, Institut Agro, Université d'Angers, IRHS, SFR QUASAV, F‐49000 Angers, France; INRAE, Institut Agro, Université d'Angers, IRHS, SFR QUASAV, F‐49000 Angers, France; University of Western Australia, Australia

**Keywords:** Abscisic acid, defence, exudate, jasmonic acid, 12-oxo phytodienoic acid, pathogenesis-related proteins, primary dormancy, secondary dormancy, seed coat, spermosphere

## Abstract

To ensure their vital role in disseminating the species, dormant seeds have developed adaptive strategies to protect themselves against pathogens and predators. This is orchestrated through the synthesis of an array of constitutive defences that are put in place in a developmentally regulated manner, which are the focus of this review. We summarize the defence activity and the nature of the molecules coming from the exudate of imbibing seeds that leak into their vicinity, also referred to as the spermosphere. As a second layer of protection, the dual role of the seed coat will be discussed; as a physical barrier and a multi-layered reservoir of defence compounds that are synthesized during seed development. Since imbibed dormant seeds can persist in the soil for extensive periods, we address the question of whether during this time a constitutively regulated defence programme is switched on to provide further protection, via the well-defined pathogenesis-related (PR) protein family. In addition, we review the hormonal and signalling pathways that might be involved in the interplay between dormancy and defence and point out questions that need further attention.

## Teaser

In the soil, dormant seeds must cope with pathogens to ensure their persistence. Here we review different defence strategies that dormant seeds rely on to allow their persistence until germination.

## Introduction

Seed dormancy is an innate property that blocks the capacity to germinate over a time period under any combination of environmental conditions that otherwise support the germination process (Finch-Savage and Leubner-Metzger, 2006; Finch-Savage and Footitt, 2017; Iwasaki *et al.*, 2022). It is an adaptive strategy for the mother plant to disperse its progeny in seasonal/unpredictable environments by avoiding germination in unfavourable conditions, thereby enhancing seedling survival. Seed dormancy is divided into five categories based on the biological mechanisms and environmental cues necessary for germination (Baskin and Baskin, 2004, 2021). Physiological dormancy is found in seeds that require specific environmental cues such as temperature, light (or absence thereof), or nitrate to germinate. Morphological dormancy refers to seeds with underdeveloped embryos at dispersal, needing further growth before germination. Morphophysiological dormancy combines features of physiological and morphological dormancy, requiring both embryo maturation and environmental cue to trigger germination. Physical dormancy is due to the presence of a tough, water-impermeable seed coat that prevents moisture uptake and gas exchange and for which physical disruption is necessary for germination. Lastly, combinational dormancy involves seeds with both hard seed coats and internal physiological inhibitors, needing multiple signals to initiate germination (Baskin and Baskin, 2004, 2021). This review focusses on seeds with physiological and physical dormancy. Physiological dormancy can be subdivided in primary and secondary dormancy depending on the chronological order in which they are acquired. Primary dormancy is induced in the mother plant during seed development, then gradually lost over time after shedding during dry storage, a process called after-ripening (Finch-Savage and Footitt, 2017; Iwasaki *et al.*, 2022). In the soil seed bank, imbibed seeds that do not germinate can enter into a so-called secondary dormancy upon prolonged unfavourable environmental conditions (Finch-Savage and Footitt, 2017). Subsequently, seasonal fluctuations in environmental conditions in the soil will release and re-impose dormancy, a process known as dormancy cycling (Finch-Savage and Footitt, 2017). As a result, seeds can remain alive for years, even decades, in the soil bank whilst being in a dormant state.

In the soil, dormant seeds are obviously exposed to a range of fungal and bacterial pathogens and to predators such as nematodes, insects, and rodents. Therefore, to ensure their vital role in disseminating the species, seeds must have developed adaptive strategies to protect themselves by synthesizing defences against pathogens and predators. Defence is a generic and complex term that has received different definitions. Here we define seed defence as the strategies and mechanisms seeds use to protect themselves from predation, environmental stress, and microbial attack. Defences can be constitutive or induced by elicitation. In seeds, constitutive defences are synthesized in a developmentally regulated manner during development. It is less known whether constitutive defences are also switched on during imbibition, in association with primary and secondary dormancy.

To experimentally demonstrate that adaptive strategies exist that link seed dormancy to defences is not a trivial issue. In the vast majority of molecular studies, seeds are systematically sterilized before imbibition. Yet, in our hands, seeds of *Medicago truncatula* and tomato that are highly dormant remained surprisingly absent of fungi during incubation of several months without any prior sterilization (Bolingue *et al.*, 2010; Hubert *et al.*, 2024). The same seeds that lost viability after storage are rapidly infected by an array of microorganisms including moulds that were apparently present on the seeds but for which growth was being repressed while seeds were dormant. So far, most of our current knowledge regarding the importance of defence mechanisms in relation to dormancy comes from various ecological studies. The optimal defence theory assumes that defences incur costs because they redirect resources from growth (reviewed by Meldau *et al.*, 2012). Thus, tissues that are most valuable in terms of fitness and have the highest probability of attack are generally the best defended. This hypothesis has recently been experimentally proven for leaves (Hunziker *et al.*, 2021). Since seeds are of high value for the plant due to their vital role in dispersing the species, they represent a primary target to allocate resources to synthesize defence compounds. However, so far the optimal defence theory has not yet been demonstrated in seeds in relation to dormancy. In addition to utilizing resources to mount a defence, the developing seeds also accumulate storage reserves which require nutrients from the mother plant. How the mother plant and/or the developing seeds partition the resources between defences and development is not known.

Seed defence has been investigated mainly in relation to seed persistence in the soil (Davis *et al.*, 2008; Tiansawat *et al.*, 2014; Pollard, 2018). In their review, Long *et al.* (2015) argue that the persistence of seeds in the field depends on a complex set of interactions between mechanisms that confer resistance against ageing (i.e. dormancy and longevity) and synthesis of defences against predation, pathogen infection, or microbial decay. Unravelling these interactions in the field has proven difficult: the different sources of mortality characterizing persistence are not easy to identify and assess (e.g. seedling death), and additional factors such as seed size and shape together with environmental conditions can influence the behaviour of the predator (reviewed in Dalling *et al.*, 2020). Using an analogy with whole plant defences, Dalling *et al.* (2011, 2020) proposed that the different types of dormancy are associated with different types of defences. PY is linked to physical defences that form a barrier to exclude pathogens or to prevent the leakage of molecules that could give cues to predators regarding the presence of seeds. Seeds with physiological dormancy present a panoply of (bio)chemical defences. Seeds can also germinate rapidly as an escape strategy (Dalling *et al.*, 2011, 2020).

In this review, we will focus on the constitutive defence mechanisms that might contribute to the survival of dormant seeds in the soil. First, the nature of the defence mechanisms and molecules will be described for two protective layers: the spermosphere, representing the immediate vicinity outside the seed, and the seed covering layers including the seed coat (or testa), and in the case of indehiscent fruits the seed coat plus the pericarp (fruit walls). Next, we will address whether imbibition of dormant seeds leads to the activation of a defence programme or if the defence compounds found in the exudates originate from a build-up during seed maturation, via the well-defined pathogenesis-related (PR) protein family. In addition, we will highlight questions related to the role of jasmonic acid (JA) and abscisic acid (ABA) signalling in defence in seeds that need further attention to obtain a better understanding of how seeds defend themselves.

## Exudation into the spermosphere

**Fig. 1.**
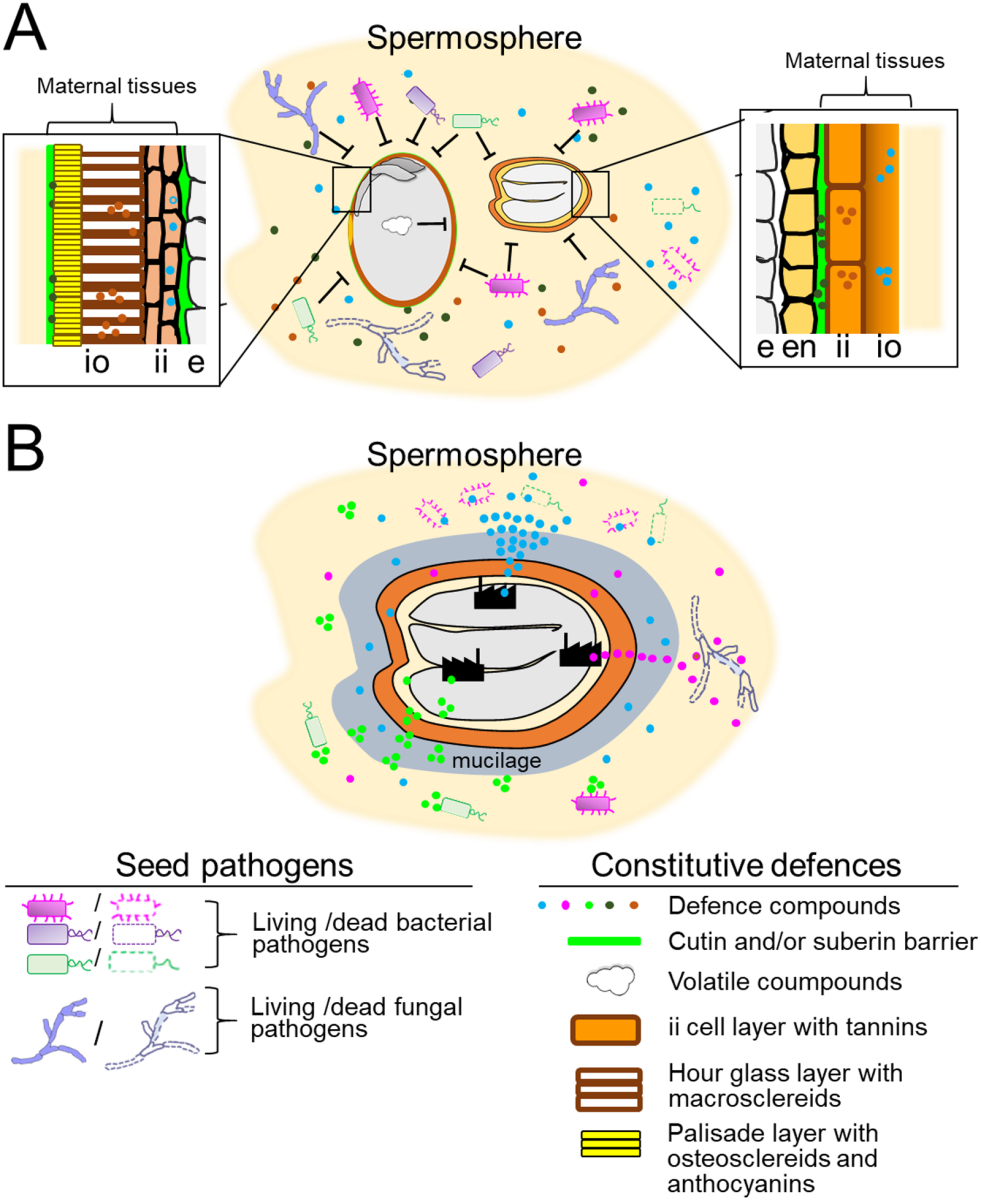
Schematic drawing illustrating the distribution of defence mechanisms in and around the seed of a representative of a legume (left, soybean) and *Brassicaceae* (right, Arabidopsis). The picture and its contents are not made to scale and the morphology of the seed coat integuments is simplified to better visualize the different components of seed defence. (A) In cases of physical dormancy (soybean) and physiological dormancy (Arabidopsis), the seed coat provides most of the protection by leaking defence compounds that are stored in the teguments into the spermosphere. In addition, the cell walls reinforced by condensed tannins (PA) and lignin, and the presence of impermeable layers (cutin and suberin) impede both entry of pathogens and release of volatile compounds produced by the embryo. (B) An Arabidopsis seed exhibiting primary or secondary dormancy during prolonged imbibition that leads to mucilage formation. The embryo and/or endosperm continuously synthesize compounds with antibacterial and antifungal activities whereas the mucilage and seed coat provide an additional barrier for the pathogens. Abbreviations: e, embryo; en endosperm; ii, inner integuments, oi outer integuments.

In the soil seed bank, dormant seeds are subject to multiple dehydration/rehydration cycles. During water uptake, they exude a variety of molecules including defence compounds outside in their vicinity, thereby impacting the spermosphere and providing thus a first layer of protection (Fig. 1). Schiltz *et al.* (2015) defined the spermosphere as the region of soil directly under the influence of seeds, serving as a crucial interface for both advantageous and harmful interactions between seeds and microbes. The timing of exudation of defence molecules during imbibition is suggested to occur in two phases (Schiltz *et al.*, 2015). First, upon imbibition, the seed undergoes a swift expansion, altering both its size and shape, leading to a transient structural disturbance in the cellular membranes resulting in an immediate and rapid release of solutes and low molecular weight metabolites into the surrounding imbibition solution. A second burst occurs at the end of germination when the radicle protrudes through the seed coat. Once the radicle emerges, the soil environment surrounding the seed is defined as the rhizosphere. The spermosphere has thus a temporary existence during germination. Yet, considering that dormant seeds may remain hydrated in the soil for prolonged amounts of time, this temporal nature of the spermosphere will be extended.

Little is known about the specific nature of the exudates from dormant seeds, and whether its composition and the antimicrobial properties are similar to that of seeds before and after radicle emergence. Hubert *et al.* (2024) demonstrated that antimicrobial activity of the exudate of primary dormant tomato seeds, measured by the growth reduction of the fungus *Alternaria brassisicola*, becomes detectable only after 3 d imbibition and continues to increase upon further imbibition (Hubert *et al.*, 2024). This implies that leakage of the defence molecules that conferred the antimicrobial activity is not instantaneous upon initial water uptake. This raises the intriguing question of whether these molecules were already present in dormant seeds but that they are extruded in a time-dependent, active manner, or whether they are being re-synthesized during imbibition. Furthermore, exudation of antimicrobial compounds was also detected in seeds in which secondary dormancy was induced, suggesting that such a defence mechanism is also associated with dormancy cycling (Hubert *et al.*, 2024).

**Fig. 2.**
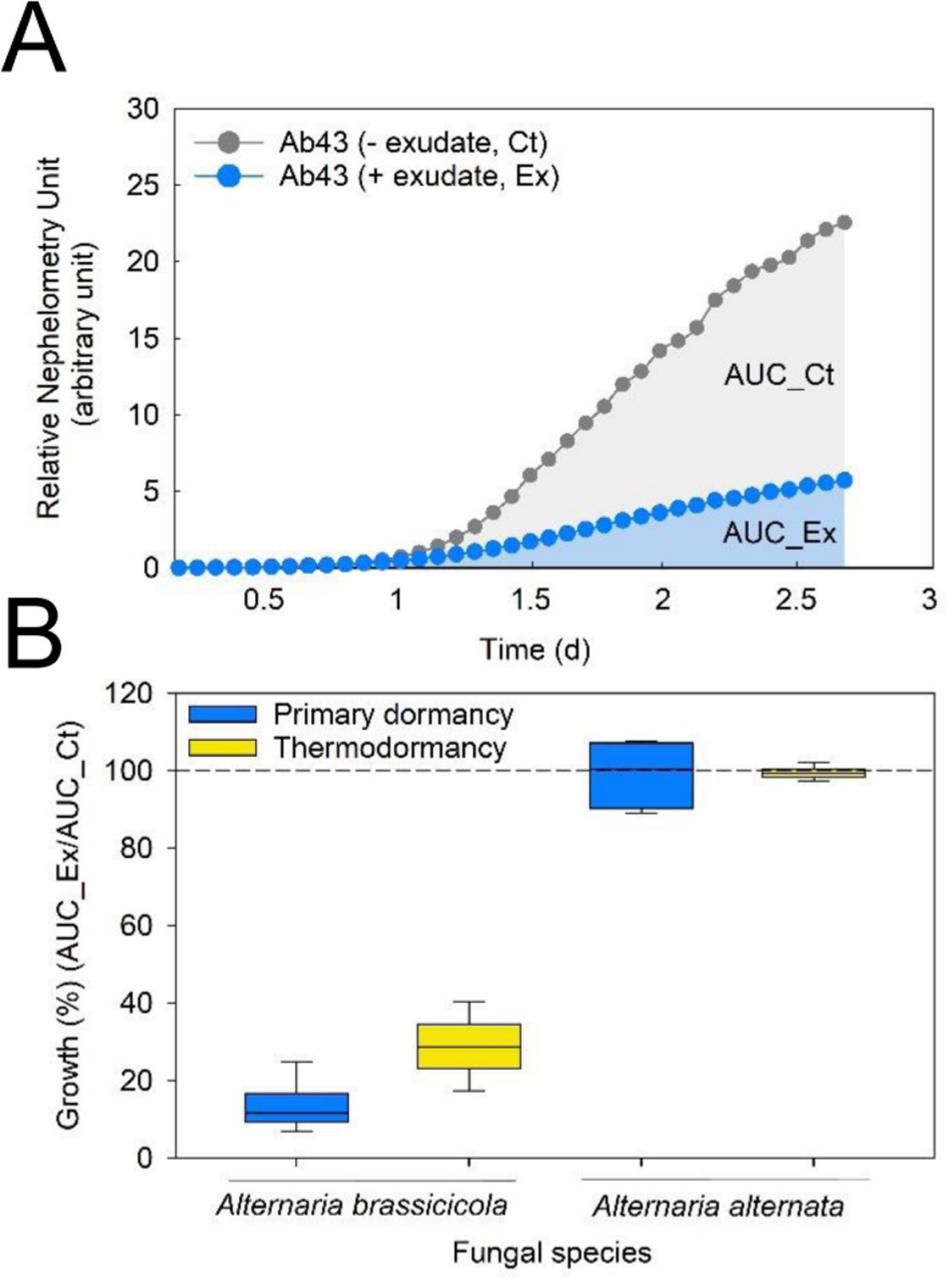
Exudate from dormant tomato seeds exhibit antifungal activity according to the nature of the pathogen. The antifungal activity was determined using a nephelometry assay. (A) Growth curve of *Alternaria brassisicola* (strain Ab43) at 10^3^ colony forming unites (CFU) ml^–1^ with (+ exudate, Ex) and without (- exudate, Ct) exudates that were collected from primary dormant seeds during the first 5 d of imbibition. The areas under the curve (AUC) are indicated (in blue for AUC_Ex). (B) Exudates from primary and thermodormant tomato seeds display antifungal activity for Ab43 but not for *A. alternata* (strain NB100 at 10^3^ CFU ml^–1^), a pathogenic fungus of the *Solanaceae* family. Thermodormant seeds were obtained after 5 d imbibition at 35 °C. Antifungal activity is expressed as the growth ratio between the AUC with (AUC_Ex) and without (AUC_Ct) exudates. The dashed line corresponds to control growth without exudate. Abbreviations: AUC, area under the curve; Ct, control, Ex, exudate. Data are redrawn from Hubert *et al.* (2024).

To obtain an overview on the nature of the defence molecules that are part of the exudate, we searched the literature for studies that provide experimental evidence of antimicrobial or antipredator activity of the seed exudate, restricted to studies on exudates coming from seeds prior to germination. An overview of these studies is presented in Table 1, together with the composition of the molecules that was found in the exudates, when available. Although only two studies mentioned that the exudate came from seeds that were dormant (Fuerst *et al.*, 2018; Hubert *et al.*, 2024), it is evident that exudates from imbibing seeds can possess a wide defensive arsenal with antifungal, antibacterial, nematicidal, antihelminthic, and antiviral activities (Table 1). Interestingly, exudates from maize and black bean seeds were found to contain unidentified proteins that exert different inhibitory effects on *Phytophthora sojae (**Zhang et al., 2022**).* In maize, in which this soilborne pathogen does not infect the seed, the exudate proteome contains repellent peptides that are sensed by the zoospores and strongly inhibit chemotaxis signals. In bean seeds, for which *P. sojae* is a pathogen, the exudate contains molecules that dissolve the cysts (i.e. the resting zoospores) (Zhang *et al.*, 2022). In a similar way, the exudate from seeds can have a different effect according to the nature of the pathogen. For instance, exudates from tomato seeds can repress the growth of *A. brassisicola* (a pathogen for *Brassicaceae*), but not of *Alternaria alternata*, a pathogenic fungus of the *Solanaceae* family, showing that the antimicrobial activity is non-host specific (Fig. 2; Hubert *et al.*, 2024).

**Table 1.** Defence activity of seed exudates from ungerminated imbibed seeds

Type of activity	Microorganism	Plant species	Molecules present in exudate	Publication
**Antifungal**	*Leptosphaeria* sp.	*Macadamia integrifolia*	Peptides from vicilin 7S	Marcus *et al.*, 1999
	*Cercospora kikuchii*	*Glycine max*	Soybean toxin (SBTX)	Arantes *et al.*, 2020
	*Fusarium oxysporum* f. sp*. melonis*	*Anastatica hierochuntica* (seed coat)	Antifungal proteins, nucleases, proteases and chitinases	Raviv *et al.*, 2017
	*Colletotrichum graminicola*	*Kochia scoparia*	NA^*a*^	Houlihan *et al.*, 2019
	*Fusarium avenaceum* (resistant to seed decay)	*Triticum aestivum* L. (leachated primary dormant caryopses)	Polyphenol oxidase, exochitinase, peroxidase, oxalate oxidase	Fuerst *et al.*, 2018
	*Alternaria brassicicola*	*Solanum lycopersicum* (primary/secondary dormant)	NA^*a*^	Hubert *et al*., 2024
	*Phytophthora sojae*	*Phaseolus vulgaris*	NA^*a*^	Zhang *et al.*, 2022
**Antibacterial**	*Staphylococcus aureus*	*Anastatica hierochuntica* (seed coat)	Antifungal proteins, nucleases, proteases, and chitinases	Raviv *et al.*, 2017
	*Rhizobium meliloti*	*Medicago sativa*	NA^*a*^	Jain and Nainawatee, 1999
**Nematicidal**	*Heterodera schachtii, Meloidogyne hapla* and *Pratylenchus penetrans*	*Tagetes erecta*, *Tagetes patula*	NA^*a*^	Riga *et al.*, 2005
	*Meloidogyne incognita*	*Glycine max*	β-1,3-Glucanase, chitinase, lectin, trypsin inhibitor, and lipoxygenase	Rocha *et al.*, 2015
	*Meloidogyne incognita*	*Moringa oleifera*	β-1,3-Glucanases, chitinases, proteases; serine and cysteine protease inhibitors	Sousa *et al.*, 2020
**Anthelminthic**	*Haemonchus contortus*	*Myracrodruon urundeuva*	Protease inhibitor, peptidase, chitinase, lipases, ellagic acid, quercetin, rhamnoside	Soares *et al.*, 2018
	*Haemonchus contortus*	*Glycine max*	NA^*a*^	Ribeiro *et al.*, 2021
**Antiviral**	Rabies virus	*Phaseolus vulgaris*	Anthocyanin-related substance	Kawai and Fujita, 2007
**Chemotaxis alteration**	*Phytophthora sojae*	*Glycine max*	NA^*a*^	Zhang *et al.*, 2020
	*Phytophthora sojae*	*Zea mays*	NA^*a*^	Zhang *et al.*, 2022

^*a*^ NA, Information not available.

For those studies that identified putative molecules that could be underlying the defence activity, many focused on the analysis of the proteome (Table 1). Numerous proteins were detected that belong to the pathogenesis-related protein (PR) family (Tables 1, 2). PR proteins have been identified and categorized into 17 families based on shared amino acid sequences, serological relationships, and enzymatic or biological activity (Van Loon and van Strien, 1999; Sels *et al.*, 2008; Gorjanović, 2009; Planes *et al.*, 2015; Table 2). The majority of PRs possess antimicrobial activity, whereas PR-8 and PR-11 are classified as endochitinases and PR-15 and PR-16 are oxalate oxidase and oxalate oxidase- like proteins, respectively (Pollard, 2018; Li *et al.*, 2020; dos Santos and Franco, 2023). The seed exudates were shown to contain almost all of the PR families, such as PR-2 (β-1,3-glucanase), PR-3/4/8/11 (chitinases), PR-6 (protease inhibitors), and PR-14 (lipid transfer proteins) (Table 1). PR-9 (peroxidase) and PR-15 (oxalate oxidase) were found in leachates of wild oat and wheat caryopses (Fuerst *et al.*, 2018). Many PR proteins are synthesized with an N-terminal signal peptide determining translocation into the endoplasmic reticulum, followed by secretion into the apoplast (Van Loon *et al.*, 2006). Other PR proteins have additional extensions specifying deposition into the vacuole. Different members of the same PR family may or may not be secreted. For instance, for the PR-2 family (β-1,3-glucanases), Class I proteins are vacuolar and the acidic Class II and III proteins are secreted (Leubner-Metzger and Meins, 1999). PR-10-type proteins are the only family of which all members seem to be cytoplasmic. It is noteworthy that β-1,3-glucanases have a dual function in seeds, at least in the *Solanaceae*: acting as part of a pre-emptive strategy to protect a germinating seed against microbial attack and contributing to germination by promoting endosperm rupture to ensure protrusion of the radicle out of the seed coat (reviewed by Leubner-Metzger, 2003). In addition to PR proteins, the seed exudates also contain other defence-related proteins such as cystatins, lipases, trypsin inhibitors, and seed storage proteins such as vicilin-like 7S globulins that can exhibit antimicrobial activity (Rose *et al.*, 2006; de Souza Cândido *et al.*, 2011).

**Table 2.** Overview of PR protein families and their estimated size

Family	Properties	Typical size (kDa)
PR-1	Antifungal	15
PR-2	β-1,3-Glucanase	30
PR-3	Chitinase types I-II, IV-VII	25–30
PR-4	Chitin binding proteins	15–20
PR-5	Thaumatin-like protein (TLP)	25
PR-6	Protease inhibitor (PI)	8
PR-7	Endoproteinase	75
PR-8	Chitinase type III	28
PR-9	Peroxidase	35
PR-10	‘Ribonuclease-like’	17
PR-11	Chitinase, type V	40
PR-12	Defensin	5
PR-13	Thionin	5
PR-14	Lipid-transfer protein (ns-LTP)	9
PR-15	Oxalate oxidase	20
PR-16	‘Oxalate oxidase-like’	20
PR-17	Unknown	27

Data from Sels *et al*. (2008) and Gorjanovicz (2009).

Few studies have analysed the metabolome of seed exudates prior to germination. Kawai and Fujita (2007) identified specialized metabolites displaying antimicrobial activity, such as ellagic acid, quercetin, rhamnoside, and anthocyanin-related substances. In contrast, the presence of specialized metabolites has been extensively studied on exudates from the rhizosphere, after the seeds have germinated (van Dam and Bouwmeester, 2016). Considering the numerous specialized molecules that are present in the seed coat with antimicrobial properties, it is likely that more will be discovered upon analysis of exudates from dormant seeds (Corso *et al.*, 2020, 2021). The identification of defence molecules in the seed exudates with antimicrobial properties will aid the development of new bio-sourced seed treatments against soil pathogens. Our recent work on tomato shows that there is also a genetic diversity in the level of antimicrobial activity of exudate coming from dormant seeds (Hubert *et al.*, 2024). This genetic variation needs to be taken into consideration in studies on the composition and antimicrobial activity of the exudate of dormant seeds to fully exploit the beneficial role in protecting the seed from its immediate environment.

## Contrasting germination strategies resulting from the spermosphere

The spermosphere does not only contain compounds from the seed exudate, but also molecules that result from microbial activity that can influence the physiology of the seed and as such contribute to its survival in the soil. For instance, seeds have evolved an escape strategy as a defence against pathogens. *Pyrenophora seminiperda* (a necrotrophic seed pathogen) attacks primary and secondary dormant seeds of *Bromus tectorum* (a winter annual weed), penetrating directly through the seed coverings and then the endosperm, producing toxins that eventually kill them (Beckstead *et al.*, 2007; Finch-Boekweg *et al.*, 2013). Seeds are only able to survive by rapidly germinating in autumn when dormancy is released, whereas the pathogen is most effective in summer and winter when seeds are dormant. Another strategy is the elaborate germination arrest control mechanism in response to biotic factors. Rhizosphere associated strains of *Pseudomonas*, *Burkholderia*, and *Streptomyces* spp. synthesize so-called ‘germination-arrest factors’ that have been identified as different compounds of the oxyvinylglycine family (Banowetz *et al.*, 2008; Halgren *et al.*, 2013; Lee *et al.*, 2013; Chahtane *et al.*, 2018; Zhou *et al.*, 2022). This family includes, for example, rhizobiotoxin, L-2-amino-4-methoxy-trans-3-butenoic acid and aminoethoxyvinylglycine. During imbibition, these molecules chemically interfere with 1-aminocyclopropane-1-carboxylate synthase and thereby affect ethylene production in rice (*Oryza sativa*) seeds (Zhou *et al.* 2022) or DELLA activity in Arabidopsis (Chahtane *et al.*, 2018). In both species, the presence of these germination arrest factors results in the accumulation of ABSCISIC INSENSITIVE 5 (ABI5) and induction of genes associated with seed maturation and germination arrest. These observations raise an intriguing question in respect to defence and seed dormancy. Does prolonged exposure to oxyvinylglycine molecules lead to induction of secondary dormancy, comparable to the short incubation at high temperature that leads to germination arrest, whereas longer incubation time induces thermodormancy? This question is essential because the outcome could demonstrate that by detecting these molecules in the spermosphere, the seeds interpret the presence of microbes as a threat leading to ABA-induced dormancy as a defence against soilborne pathogens as speculated by Chahtane *et al.* (2018).

## The role of the seed covering layers in seed dormancy and defence

A second line of defence is constituted by the fruit/seed coat and the endosperm that represent sophisticated defence structures. In angiosperms, seed development is initiated after a double-fertilization event, which produces the endosperm and the zygote. The endosperm is triploid, bearing two maternal genomes and one paternal genome, whereas the zygote is diploid, bearing one maternal genome and one paternal genome. The seed coat is of maternal origin and results from the development of the two integuments (inner and outer) of the fertilized ovule in many different layers whose structure and composition vary from species to species (Fig. 1; Smýkal *et al.*, 2014). The seed coat is developmentally transitory as a number of tissues that present early during seed development do not persist in mature dry seeds. In addition, in species with indehiscent fruits, the pericarp, also of maternal origin, surrounds the seed coat. These tissues have a dual function in terms of protection: they provide a reservoir with a finite amount of defence compounds and proteins that become part of the seed exudate during imbibition, and they constitute several barriers to avoid pathogen entry via biochemical and morpho-physical features.

In wild *Poaceae*, the caryopsis (i.e. the fruit containing a single seed) is enclosed by the husks including the dead floral bracts, the lemma, palea, and glume that contain defence molecules (reviewed by Grafi and Singiri, 2022). These remnants of the floral tissues act as the first line of defence. Dead husks of wild emmer wheat were found to contain hundreds of proteins with the lemma and palea containing nucleases and chitinases, whereas the glume released 1-type endonucleases that had antimicrobial activity, and contained PR 1–1, PR-1-5, and PR-4 as well as antifungal hydrolases including chitinases and β-1,3-glucanase (Raviv *et al.*, 2017). A review by Pollard (2018) summarizes current knowledge on the enzyme-based biochemical seed defences (chitinase, polyphenol oxidase, peroxidase, and oxalate oxidase). Raviv *et al.* (2017) demonstrated that these enzymes are well preserved for decades in the dry state in dead tissues and become active upon imbibition. In their seminal work, Jerkovic *et al*. (2010) identified distinct protein profiles with specific defence properties according to three bran fractions in cultivated wheat, namely the outer layer (the outer pericarp), the intermediate layer (the inner pericarp and seed coat), and the inner layer (aleurone layer of the endosperm). They demonstrated that the outer layer contains enzymes such as oxalate oxidase (PR-15) and polyphenol oxidase that provide resistance against fungal and bacterial colonization, whereas the inner layer contains various PR proteins and inhibitors of enzymes secreted by pathogens such a xylanase inhibitor and an α-amylase/subtilisin inhibitor. They concluded that differential protein complements of each bran layer in wheat provide distinct lines of defence (Jerkovic *et al.*, 2010).

Most of the PR protein families and other defence polypeptides and enzymes are deposited during seed development in order to play a role during imbibition (Jerkovic *et al.*, 2010; Raviv *et al.*, 2017; Fuerst *et al.*, 2018). During seed filling, as the seed expands in volume, some of the cellular layers will be crushed together and/or disappear, whereas later on during maturation drying, the loss of water will induce the death of the tissues, thereby sealing the fate as to how imbibing seeds will be able to defend themselves. For example, in the developing seed of tomato, seed coat-specific transcript profiles exhibited an over-representation of defence related genes at the onset of seed filling (Bizouerne *et al.*, 2021). During seed development of *Brassicaceae*, the seed coat also ensures the loading of glucosinolates whose biosynthesis is restricted to maternal tissues into the embryo where these defence compounds accumulate (Sanden *et al.*, 2024).

Cellular and biochemical means to modulate seed coat permeability are amazingly diverse among seed species. Complete seed coat impermeability that results in PY or seed hardness by preventing seed hydration and oxygen diffusion is the ultimate effective physical barrier against pathogens (Baskin and Baskin, 2021). It is caused by the presence of one or more water-impermeable layers of palisade cells in the seed coat or pericarp and a range of polymeric compounds (Fig. 1) and we refer the reader to excellent reviews on the factors contributing to PY (Smýkal *et al.*, 2014; Huss and Gierlinger, 2021). It is evident that this trait plays an important role in seed defence and persistence in the soil, as reviewed by Dalling *et al.* (2020). For instance, morphological features modifying seed coat permeability correlate with the resistance of certain soybean cultivars to infection by *Phomopsis phaseoli* (Kulik and Yaklich, 1991). Seed coat of cultivars with low *P. phaseoli* infection lacked pores and had a closed micropyle, whereas those with high infection had multiple pores and an open micropyle. Another advantage of an impermeable seed coat is to prevent volatile compounds from escaping the seeds, which reduces detection by predators (Paulsen *et al.*, 2013).

For those seeds that do not exhibit PY, the integrity or permeability of the integument layers also play a dual role in the seed protection against pathogens while regulating physiological dormancy. For example, in peanut, removal of the seed coat greatly increases colonization of the seed by *Aspergillus flavus* (Commey *et al.*, 2021). Lipid polyesters such as cutin and suberin restrict pathogen entry in the tissues of non-seed systems (Chen *et al.*, 2022). In Arabidopsis, where both barriers are found, seeds from mutants deficient in key genes involved in their synthesis exhibit low dormancy (Giorgi *et al.*, 2015; Fedi *et al.*, 2017). It would be interesting to investigate whether they are more susceptible to pathogen attack. Such layers of lipid polyesters contain additional hydrophobic molecules with antifungal properties, acting also as reservoir of defence compounds. For example, in the barley (*Hordeum vulgare*) seed coat, the epicuticular waxes contain 5-(n)-alkylresorcinols that provided resistance against *Aspergillus* and *Penicillium*, showing a good correlation between their concentration amongst different cultivars and seed infection (García *et al.*, 1997). In cereals, these compounds accumulate specifically in the outer cuticle of the testa and the inner cuticle of the pericarp, reinforcing the idea that seeds distribute their defence compounds in distinctive layers (Landberg *et al.*, 2008). Next to lipid polyesters, phenolic compounds (or phenylpropanoids) such as anthocyanins and proanthocyanidins (PA or condensed tannins) contribute to decreased permeability of the seed coat layers and dormancy. Indeed, a suite of *transparent testa* (*tt*) mutants deficient in flavonoid synthesis, precursors of PA, produce colourless seeds with reduced dormancy and longevity (Debeaujon *et al.*, 2000; Clerkx *et al.*, 2004). Using reciprocal crosses with a seed colour gene conferring resistance to *Globisporangium ultimum* (formerly *Pythium ultimum*), Ewing *et al.* (2024) elegantly demonstrated that the seed resistance is conferred by soluble and insoluble PA that were associated with the testa and not the embryo or the colour of the testa. They showed that insoluble PA was associated with thick-walled sclerenchyma acting as a physical and impermeable barrier against the pathogen, whereas the soluble PA eluted into the spermosphere to inhibit fungal growth. Likewise, seeds of several PA-free barley mutants were unable to stop the growth of several *Fusarium* species within the testa (Skadhauge *et al.*, 1997). Interestingly, seeds of a mutant over-accumulating small amounts of the water soluble flavonoid dihydroquercetin, as a result of nonsense mutations in the structural gene for dihydroflavonol reductase, were highly resistant, and this flavonoid proved to be a strong inhibitor of *Fusarium* growth (Skadhauge *et al.*, 1997). Altogether, these observations reinforce the concept of a multi-layered defence mechanism where soluble PA is stored in a reservoir within the seed coat layers and polymerized to form physical barriers. For a detailed list of phenolic compounds that accumulate in seeds and the regulators of their biosynthesis, we refer the reader to Corso *et al*. (2020, 2021).

The maternal environment, such as temperature, light, and nutrients, has a strong influence on dormancy, with temperature being the most important factor (Iwasaki *et al.*, 2022). Cold temperatures during seed development generally promote dormancy. Interestingly, in Arabidopsis they also increase the expression of phenylpropanoid synthesis genes, leading to higher concentrations of procyanidins in the seed coat and higher permeability (MacGregor *et al.*, 2015). A similar scenario was found for the seed coat suberin, where the composition varies with environmental temperature during seed maturation (Fedi *et al.*, 2017). In seeds of mutants defective in the biosynthesis and transport of fatty acids for suberin deposition, dormancy lost its sensitivity to cold temperature and the seed coat exhibited an increased permeability. How environmental factors regulate the final anatomy and composition of the seed coat in relation to dormancy and resistance against pathogens remains unknown. Together these observations warrant further investigation on whether the seed has co-opted defence mechanisms from vegetative tissues that modulate seed coat properties and dormancy as an adaptive strategy to ensure the seedling establishment in unpredictable environments, including the presence of pathogens and predators. There is also the possibility that seed endophytes can participate in the synthesis of defence compounds. The beneficial effects of endophytes on seed vigour has recently been reviewed by Rétif *et al.* (2023). Although leaf endophytes have been demonstrated to produce antimicrobial compounds, research in seeds is in its infancy.

Another example of sophisticated multi-layered protection against pathogens are specialized morphological features at the seed coat surface. Seed coat cells can differentiate into epidermal hair-like structures comparable to trichomes. Defence-related compounds such as the glycoalkaloid α-tomatine accumulate in tomato leaf trichomes and this might also be the case for seed coat hairs (Balcke *et al.*, 2017; Bednarz *et al.*, 2019). Other structures that are thought to contribute to defence are cells that upon imbibition produce a mucilage. This is a complex assembly of polysaccharides, which can include pectic, cellulosic, and hemicellulosic sugars, and whose composition, architecture, and quantity vary from species to species (Tsai *et al.*, 2021). Upon imbibition, these polysaccharides rapidly expand after breaking the outer primary cell walls to form a thick mucilage capsule around the seed. As the mucilage serves as the boundary between the seed and its surroundings, it is probable that it plays a role in shaping interactions between the seed and various organisms in the spermosphere. For instance, in basil seeds, mucilage has the capacity to concentrate phenolic compounds on the seed surface, potentially providing protection against pathogens (Lee *et al.*, 2020). Mucilage might also play a role in protecting the seed from predators such as ants, in which mucilage is thought act either as a physical deterrent or a chemical repellent to discourage harvesting by ants (LoPresti *et al.*, 2019).

## A peek into constitutive defence programmes in dormant seeds via expression of members of the pathogenesis-related protein family

As indicated in the previous sections, the seed coat and exudate from imbibing seeds contain many defence compounds, including members of most of the PR protein families. Whereas the synthesis of these molecules is developmentally regulated, the question remains whether during imbibition, the dead pericarp and/or seed coat layers will simply serve as a reservoir from which defence compounds leak (Raviv *et al.*, 2017; Grafi and Singiri, 2022), or whether a constitutive defence is programmed during imbibition in dormant embryos and/or living endosperm. There is some evidence for the existence of a transcriptional programme that is turned on during imbibition in dormant seeds of *M. truncatula* (Bolingue *et al.*, 2010). A transcriptome study on seeds that were either freshly harvested (dormant) or after-ripened for 6 months (non-dormant) revealed that transcripts related to genes involved in biosynthesis of the phytoalexin medicarpin and the PR-10 proteins were much more strongly up-regulated during imbibition in dormant seeds compared to non-dormant seeds (Bolingue *et al.*, 2010). Additional evidence for the activation of a defence programme comes from the comparison between transcriptomes of the micropylar and chalazal endosperm from imbibing dormant and after-ripened, non-dormant seeds in Arabidopsis (Dekkers *et al.*, 2016). Gene expression profiles that were up-regulated in dormant compared to non-dormant tissues were enriched in gene ontology (GO) terms related to biotic stresses (defence response, immune response, response to chitin). Two other interesting GO terms related to defence are ‘jasmonic acid biosynthesis process’ and ‘response to salicylic acid stimulus’. More recently, a transcriptomic study of imbibition on dormant and non-dormant blackgrass (*Alopecurus myosuroides*) seeds also revealed an increase in the expression of defence genes in dormant tissues, such as chitinase, the xylanase inhibitor protein, and the PR-12 protein defensin 1 (Holloway *et al.* 2024).

To further investigate the hypothesis of transcriptional activation of a defence programme during imbibition, we data mined a previously published dataset on transcriptomes from imbibed dormant seeds from Cadman *et al.* (2006). As a proxy of the defence programme, we used genes encoding the PR protein family (Table 2). Although the definition of PR proteins is ‘inducible defence-related proteins’ (Van Loon *et al.*, 2006), their expression can also be developmentally regulated. We did not include the PR-7 genes which encode endopeptidases that have many different functions that are not necessarily associated with defence and represent a very large family of 27 members.

**Fig. 3.**
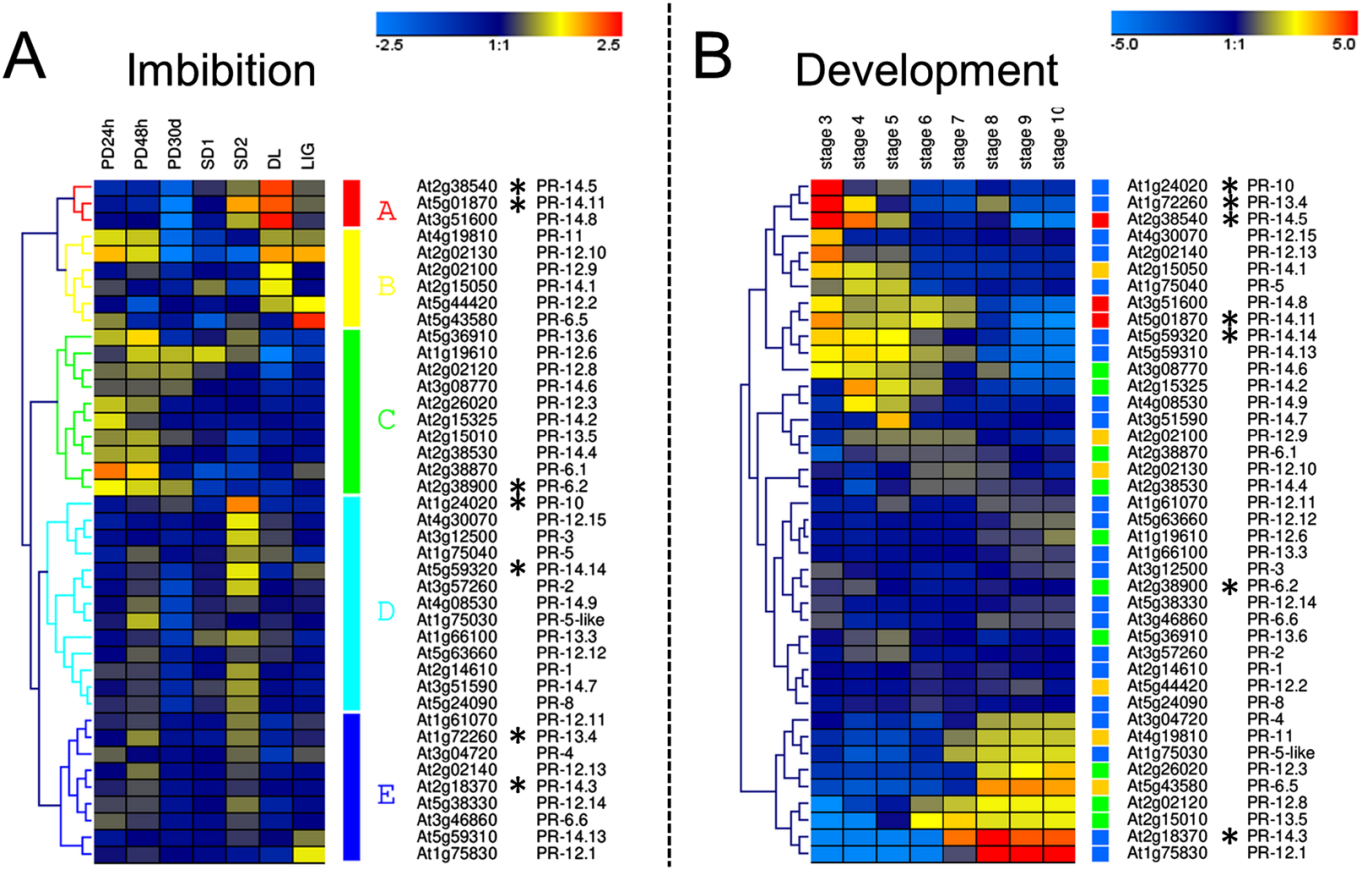
Changes in pathogenesis-related transcript levels suggest that seeds activate transcriptional defence programmes in Arabidopsis (Cvi) during imbibition in relation to dormancy status (A) and during development (B). (A) Transcript levels from seeds displaying primary dormancy after 24 h, 48 h, and 30 d of imbibition in the dark (PD24h, PD48h, PD30d), secondary dormancy (SD1, SD2), and from non-dormant, ungerminated seeds that were obtained after 120 d of dry after ripening followed by 24 h of imbibition (DL) and an additional 4 h of red light (LIG). Data are from Cadman *et al.* (2006) and were extracted from the eFP browser (https://bar.utoronto.ca/efp//cgi-bin/efpWeb.cgi?dataSource=Seed). SD1 was obtained by incubating DL seeds for a further 24 d in the dark. SD2 seeds were obtained by incubating SD1 seeds at 3 °C for 20 d and represent a dormancy cycle. (B) Transcript levels from developing seeds harvested at the following stages: stage 3, mid-globular to early heart embryos; stage 4, early to late heart embryos; stage 5, late heart to mid-torpedo embryos; stage 6, mid- to late torpedo embryos; stage 7, late torpedo to early walking-stick embryos; stage 8, walking-stick to early curled cotyledons; stage 9, curled cotyledons to early green cotyledons; stage 10, green cotyledons embryos. Stages 3–5 represent seeds and siliques, and stages 6–10 only seeds. Data are from Schmid *et al.* (2005). Transcript levels are expressed as log_2_ values after mean gene normalization. Stars indicate enriched expression in seed coat tissues during development, taken from Le *et al.* (2010).

Cadman *et al*. (2006) generated transcriptomes from primary (PD), secondary dormant (SD), and after-ripened, non-dormant Arabidopsis ecotype Cvi seeds that were all imbibed for various amounts of time. Transcripts representing the 42 genes of the different PR families were retrieved to investigate whether they are up-regulated in association with dormancy (Fig. 3). A hierarchical clustering of the transcript levels revealed four clusters of gene expression. Clusters A and B represent genes with transcripts that were high in the after-ripened states (DL, light requiring: seeds dry after-ripened for 120 d and then imbibed for 24 h in the dark and or LIG, light induced to germinate: seeds dry after-ripened for 120 d, imbibed for 20 h in the dark and then for 4 h in red light to terminate dormancy and induce germination), and for some also present in dormant samples. These clusters contained PR family members 6, 11, 12, and 14. Cluster C represents transcripts from 10 PR genes that were expressed almost exclusively in primary dormant seeds, either within the first 48 h of imbibition or even detectable after 30 d of imbibition (Fig. 3A). Most of these genes had barely detectable transcripts in dry PD seeds [eFP browser (https://bar.utoronto.ca/efp//cgi-bin/efpWeb.cgi?dataSource=Seed), Finch-Savage *et al.* (2007)], suggesting that these transcripts appeared during imbibition. Intriguingly, these genes belong to the PR-6, 12, 13, and 14 families that are all antimicrobial peptide families (Sels *et al.*, 2008). The PR-12.6 gene (i.e. plant defensin PDF1.4) is expressed in all the dormant samples and transcripts are absent in the non-dormant samples. Cluster D contains 22 genes with transcripts that are higher in the SD2 samples that correspond to secondary dormant seeds after a subsequent cycle of dormancy breaking and are deeply dormant (Cadman *et al.*, 2006). For most of the genes in this cluster, transcripts are also detected in PD seeds after 48 h of imbibition (Fig. 3A). In this cluster, transcript levels of almost all the PR protein families increased (Fig. 3A). Whereas it remains to be determined if these transcripts are being translated, this analysis reveals that clusters of PR genes are expressed during imbibition in dormant seeds, and this seems to be specific to the type of dormancy (PD or SD). Furthermore, this study demonstrates that members from the same gene families (i.e. PR12, PR13, PR14) can have very different expression profiles (Fig. 3). An intriguing question that needs to be addressed is whether the different repertoires of PR proteins reflect specific defence needs associated with primary and secondary dormancy.

To investigate whether PR genes in cluster C and D are part of a similar regulatory module that is also activated during seed development, when dormancy is installed, data from Col-0 Arabidopsis seed development were obtained from Schmid *et al.* (2005). PR gene expression is separated in two main clusters (Fig. 3B). One cluster contains genes that are expressed between the globular and mid-torpedo stage of seed development, when the inner layer of the seed coat synthesizes PA and deposits cutin (Schmid *et al.*, 2005; Iwasaki *et al.*, 2022). A second cluster correspond to 13–17 d after pollination, at the end of seed filling and beginning of maturation drying. To visualize the correspondence between transcript levels during imbibition and seed development, the colour of the clusters from Fig. 3A is indicated next to each gene in Fig. 3B. The genes from cluster A of the imbibition transcriptomes (Fig. 3A) are all expressed early during seed development. In contrast, there is no correspondence between the clustering of the PR genes associated with dormancy (Fig. 3A) and clustering of the developmental profiles (Fig. 3B), demonstrating that they are regulated by different genetic programmes.

Since many PR proteins accumulate in the seed coat, and this tissue will die during final seed maturation, we investigated in which tissues PR genes were expressed. Using expression data from different compartments and tissues of the seed during development (Le *et al.*, 2010) we identified seven genes with seed coat-specific transcripts (stars in Fig. 3). Five of the seven genes are expressed in imbibed dormant seeds (stars in Fig. 3A), suggesting that the tissue-specific regulation of these genes is different from that seen in seed development.

The endosperm is essential to repress the germination of dormant seeds. In Arabidopsis, dormancy levels can be maternally regulated by specific genomic imprinting programmes that take place in the mature endosperm (Piskurewicz *et al*., 2016). Therefore, we addressed whether genomic imprinting could be implicated in the maternal inheritance of dormancy-associated PR genes, using a dataset originating from dormant and non-dormant F_1_ endosperms resulting from Col × Cvi and Cvi × Col crosses (Piskurewicz *et al*., 2016). Eight *PR* transcripts could be identified with allelic variance between Col0 and Cvi, with three genes (*PR-12.10*, *PR.8*, and *PR-4*) showing higher transcript levels in endosperms from dormant seeds compared to non-dormant seeds for both the Col-0 and Cvi alleles, irrespective of the direction of the cross between both genotypes. However, none of these PR genes were part of the 71 maternally-expressed genes present in dormant F_1_ endosperm from both crosses.

In dormant seeds, light can be perceived as an environmental cue that triggers release of dormancy and germination. In the case of seeds with physiological dormancy, a non-satisfied light requirement is instrumental for determining a persistent seed bank, since this condition allows the seeds to perceive the signals that would induce them into secondary dormancy, thus determining dormancy cycling. This scenario illustrates the importance of a light requirement for the formation of a persistent seed bank. Consequently, whether light would regulate a long-term protection against pathogens deserves to be addressed. One of the most evident candidates to regulate the interaction between dormancy and defence could be phytochrome interacting factors (PIFs). The role of PIFs in seed dormancy and inhibition of germination has been well documented (Zhao *et al.*, 2022), and PIFs are known to function as a signal hub that integrates multiple environmental cues, including abiotic (i.e. drought, temperature, and salinity) and biotic stresses to optimize plant growth and development (for review see Li *et al.*, 2024). In seeds, PIF1 represses germination via interaction with phytochrome B (phyB). In darkness, PIF1 accumulates and enhances ABA synthesis while inhibiting GA biosynthesis, promoting dormancy. When exposed to light, phytochromes cause the degradation of PIF1, reducing ABA levels and increasing GA, which collectively promotes seed germination. Other PIFs are also involved in inhibition of seed germination, as the quadruple mutant *pif1 pif3 pif4 pif5* seeds show significant insensitivity to ABA during seed germination (Qi *et al.*, 2020). However, whereas the role of these PIFs in defence has been established in leaves, they appear to repress the transcription of basal defence genes upon infection with *Botrytis cinerea* (Xiang *et al.*, 2020). Thus, a role for the activation of a constitutive defence response in seeds by PIFs seems unlikely. An *in-silico* analysis of *cis*-acting regulatory elements of PR proteins of Arabidopsis and rice identified a high number of light-responsive *cis*-elements (Kaur *et al.*, 2017). One of them is the G-box (CACGTG) involved in response to light, ABA, and methyl jasmonate (MeJA), and has a role in seed-specific expression. It has been shown to be present in all Arabidopsis PR genes except in *AtPR2* and in all rice PR genes. Its putative role in repressing *PR* activity in seeds needs to be investigated.

## Hormonal pathways controlling defence and seed dormancy

In contrast to the progress on deciphering the phytohormonal module controlling seed germination and dormancy (see Iwasaki *et al.*, 2022; Pan *et al.*, 2023; Sajeev *et al.*, 2024 for recent reviews), a mechanistic explanation for how phytohormones regulate the accumulation of defence molecules during maturation or imbibition in dormant seeds remains unknown. ABA is the main hormone controlling seed dormancy and germination. Repression of seed germination in imbibed dormant seeds is due to sustained high ABA accumulation, both in the embryo and endosperm, over time as a combined result of initial ABA concentration present in dry seeds and the balance between *de novo* ABA synthesis and catabolism (Finch-Savage and Leubner-Metzger, 2006; Iwasaki *et al.*, 2022; Zhao *et al.*, 2022). This activates the ABA signalling pathway via ABI3 and ABI5 to block seed germination and maintain the embryonic state. In Arabidopsis, the endosperm plays a key role in dormancy by synthesizing and exporting the ABA to the embryo and repressing its growth potential (Piskurewicz *et al.*, 2016; Iwasaki *et al.*, 2022).

**Fig. 4.**
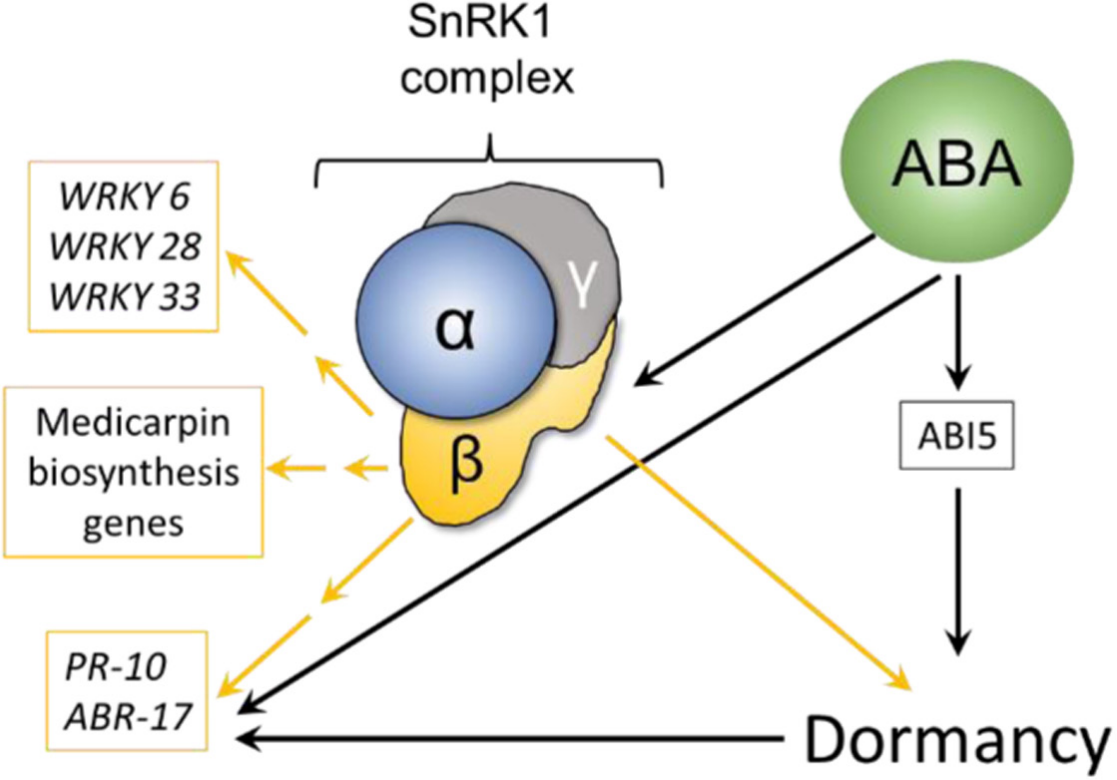
The regulatory SNF4b subunit of the SnRK1 complex at the interface between dormancy and constitutive defence in *Medicago truncatula* seeds. Model depicting the regulation of a SnRK1 kinase complex including MtSNF4b. Yellow and black arrows indicate stimulation dependent and independent of SNF4b, respectively. Defence genes are framed in boxes. Data are from Bolingue *et al.* (2010).

Direct evidence of a hormonal regulation at the interface between defence and dormancy comes from our work in *M. truncatula* for which we found that seeds from the silencing mutant of MtSNF4b, an ABA-inducible regulatory subunit of the sucrose non-fermenting-related kinase complex (SnRK1), exhibit reduced dormancy (Bolingue *et al.* 2010; Fig. 4). SnRK1 kinase complexes are involved in regulating plant immunity (Hulsmans *et al.*, 2016; Margalha *et al.*, 2019). Consistent with this, *Mtsnf4b* seeds display a reduced expression of over 120 defence genes including PR-10, most of the genes involved in the biosynthesis of medicarpin, an antifungal phytoalexin, and WRKY transcription factors involved in the defence response (Bolingue *et al.* 2010; Fig. 4). The PR-10 protein that is up-regulated during imbibition in dormant seeds of *M. truncatula* compared to non-dormant, ungerminated seeds is also known in pea as ABA-responsive 17 (ABR17, Iturriaga *et al.*, 1994). Altogether, these observations link the synthesis of constitutive defences to ABA. Three PR genes (*PR-8*, *PR-14.14*, *PR-5-like*) were found among the Arabidopsis ABI5 targets (O’Malley *et al.*, 2016; Yang *et al.*, 2023) and both *PR-5-like* and *PR-14.14* are co-expressed with *ABI5* (r=0.94 and –0.93).

In conjunction with ABA, JA might play a key role at the interface between dormancy and constitutive seed defence, considering its importance in regulating defence responses in vegetative tissues (reviewed in Li *et al.*, 2022). Whereas JA is the basic form of the hormone, the conjugated form jasmonoyl-isoleucine (JA-Ile) is the bioactive form. MeJA is a volatile ester of JA used to simulate the effects of JA. Jasmonate glucosides are conjugated forms of jasmonates with sugars, which can serve as storage or inactive forms that can be converted back to active jasmonates when needed. Studies showing that seed defences dependent on JA are scant. The possible role of JA in seed defence in dormant seeds is indirectly supported by a transcriptome analysis of dormant Arabidopsis endosperm as mentioned above, showing an enrichment in GO terms associated with the JA biosynthetic process (Dekkers *et al.*, 2016). A closer look at genes in this GO term that were up-regulated in dormant versus non-dormant tissues identified *ALLENE OXIDE CYCLASE3* (*AOC3*) and *AOC4*, enzymes involved in JA biosynthesis, and *JASMONATE RESISTANT 1* (*JAR1*) that catalyses the formation of a biologically active jasmonyl-isoleucine (JA-Ile) conjugate. In the tomato jasmonate-deficient *defenseless1* mutant, expression of the PR class I chitinase 9 (*Chi9*) was reduced in imbibing seeds and could be restored by MeJA treatment (Wu and Bradford, 2003). In contrast to biotic stress, the role of JA in seed dormancy or germination arrest upon abiotic stress has received much more attention. JA inhibits germination during imbibition of non-dormant Arabidopsis seeds, acting upstream of ABA signalling by interacting with ABI5 (see Pan *et al.*, 2023 for a review). Likewise, JA inhibits pre-harvest sprouting during wheat seed development, also via the interaction with ABI5 (Ju *et al.*, 2019). In contrast to the inhibitory effect on non-dormant seeds, JA has the opposite effect on dormant seeds, at least in cereals. It induces dormancy release during imbibition in wheat and barley (Barrero *et al.*, 2009; Nguyen *et al.*, 2022). In wheat, this JA-induced dormancy release is mediated by an increase in seed sensitivity to GA. In a comparable manner, Arabidopsis plants that were attacked by red spider mite, an arachnid herbivore that activates JA-dependent defences, produced seeds that were not dormant in contrast to non-infected controls (Singh *et al.* 2017). The seeds from infested plants contained elevated amount of JA-Ile and GA and reduced levels of ABA compared to untreated controls. Whereas it is clear that JA induces germination arrest, whether it plays a role in the induction of constitutive defence in relation to dormancy remains to be investigated.

Downstream of JA, JASMONATE ZIM-DOMAIN (JAZ) proteins play a central role in JA signalling, acting as a repressor that needs to be removed to activate a JA response. A detailed account of the JA signalling pathway can be found in the excellent reviews by Liu *et al*. (2021), Jimenez Aleman *et al*. (2022), Li *et al*. (2022), and Pan *et al*. (2023). Different domains in the JAZ proteins serve as binding sites for sets of proteins that are specifically associated with various defence responses, creating differential modes of repression for each JAZ against a range of pathogens (Goossens *et al.*, 2015; Liu *et al.*, 2021). A similar scenario has been observed between JAZ and regulators controlling germination. In wheat, TaJAZ1 binds to TaABI5 to repress its activity and induces preharvest sprouting whereas in Arabidopsis, nine out of the 13 JAZ proteins bind to ABI5 (Ju *et al.*, 2019). Wen *et al.* (2018) showed that JAZ2 and JAZ11 interact *in vitro* with TRANSPARENT TESTA8, a transcriptional factor that acts maternally on seed development and regulates dormancy. JAZ9 binds to ARF10, ARF16, and ABI5 to enhance ABA-mediated seed dormancy, indicating that JAZ proteins also integrate signals from other hormone pathways such as auxins (Mei *et al.*, 2023). The bioactive form of JA, JA-Ile, binds to a co-receptor complex composed of CORONATINE INSENSITIVE 1 (COI1) and a JAZ protein, which is then targeted for proteasomal degradation. As a result, the repression exerted by JAZ on a panoply of transcription factors is lifted, including the master regulators MYC2 and ABI5. Mutations in MYC2 that incapacitate its binding with JAZ proteins lead to inhibition of germination due to an increased ABA hypersensitivity (Goossens *et al.*, 2015). This phenotype was also accompanied by increased transcript levels of several defence marker genes in leaves. Altogether, these observations raise the question whether within the JA-COI1 dependent signalling pathway, a seed-specific repertoire of JAZ interactions serve as an interface regulating the induction/maintenance of dormancy during seed development and/or imbibition and the synthesis of constitutive defences.

The precursor of JA, 12-oxo phytodienoic acid (OPDA), also positively regulates dormancy in synergy with ABA through ABI5 expression and stability of DELLA proteins (Dave *et al.* 2011, 2016). OPDA is actually more potent that JA because far lesser concentrations of OPDA are needed to inhibit germination. OPDA is a mobile molecule that accumulates during late seed maturation in Arabidopsis (Dave *et al.*, 2011) and could be a likely candidate to regulate defences in dormant seeds, as observed in roots and leaves [reviewed in Li *et al.* (2022) and Jimenez Aleman *et al*. (2022)].

In an effort to better decipher the roles of JA-COI1-dependent (via JAZ) and independent (via OPDA) signalling pathways in the synthesis of constitutive defence in relation to dormancy, studies at tissue level are needed. Different developmental pathways are activated in the maternal seed coat, the triploid endosperm, or the zygotic embryo, with each specific fine-tuning of hormonal and regulatory pathways. For example, during seed development, *OPR3* and *JAR1* (that produces JA-Ile) are only expressed in the endosperm. In immature bean seeds, imaging mass spectrometry detected the presence of OPDA exclusively in the hilum and seed coat, whereas ABA was distributed in the cotyledons (Enomoto *et al.* 2017). Interestingly, Dave *et al.* (2011) showed that the endosperm and seed coat rupture plays a more important role in the OPDA blockade compared to ABA which mostly inhibits embryonic growth but not testa rupture. Likewise, in Arabidopsis; the eFP browser revealed that JAZ genes are predominantly expressed in the seed coat and endosperm tissues during seed maturation, particularly *JAZ2* and *JAZ9*. During seed imbibition, *OPR3*, whose deficiency leads to dormancy, is up-regulated in the endosperm when germination is blocked by ABA or paclobutrazol, a GA biosynthesis inhibitor. Consistent with these observations, MeJA induces the expression of *Chi9* only in the micropylar endosperm during imbibition in tomato (Wu and Bradford, 2003).

## Conclusion

To cope with pathogens present in soil, dormant seeds are endowed with a panoply of defence layers that are strategically distributed in different compartments, with the seed coat playing a central role. On the one hand, it constitutes several barriers to avoid pathogen entry via biochemical and morpho-physical features. On the other hand, it provides a reservoir filled with defence compounds and PR proteins that leak into the spermosphere during imbibition, thereby constituting a first level of defence against pathogens. Using the expression of PR genes as markers, it is suggested that primary and secondary dormant seeds might not only depend on a passive unloading of defence compounds, but that they might also switch on a defence programme when they are imbibed to provide further protection. Deciphering the underlying regulatory mechanisms of seed defence will need to focus at the tissue level in a temporal fashion and investigate their integration in the different signalling pathways related to seed development.
